# A *de novo *assembly of the newt transcriptome combined with proteomic validation identifies new protein families expressed during tissue regeneration

**DOI:** 10.1186/gb-2013-14-2-r16

**Published:** 2013-02-20

**Authors:** Mario Looso, Jens Preussner, Konstantinos Sousounis, Marc Bruckskotten, Christian S Michel, Ettore Lignelli, Richard Reinhardt, Sabrina Höffner, Marcus Krüger, Panagiotis A Tsonis, Thilo Borchardt, Thomas Braun

**Affiliations:** 1 Max-Planck-Institute for Heart and Lung Research, Ludwigstrasse 43, 61231 Bad Nauheim, Germany; 2 Max-Planck Genome Centre Cologne, Carl-von-Linné-Weg 10, 50829 Köln, Germany; 3 Department of Biology and Center for Tissue Regeneration and Engineering at Dayton, University of Dayton, OH 45469-2320, USA

## Abstract

**Background:**

*Notophthalmus viridescens*, an urodelian amphibian, represents an excellent model organism to study regenerative processes, but mechanistic insights into molecular processes driving regeneration have been hindered by a paucity and poor annotation of coding nucleotide sequences. The enormous genome size and the lack of a closely related reference genome have so far prevented assembly of the urodelian genome.

**Results:**

We describe the *de novo *assembly of the transcriptome of the newt *Notophthalmus viridescens *and its experimental validation. RNA pools covering embryonic and larval development, different stages of heart, appendage and lens regeneration, as well as a collection of different undamaged tissues were used to generate sequencing datasets on Sanger, Illumina and 454 platforms. Through a sequential *de novo *assembly strategy, hybrid datasets were converged into one comprehensive transcriptome comprising 120,922 non-redundant transcripts with a N50 of 975. From this, 38,384 putative transcripts were annotated and around 15,000 transcripts were experimentally validated as protein coding by mass spectrometry-based proteomics. Bioinformatical analysis of coding transcripts identified 826 proteins specific for urodeles. Several newly identified proteins establish novel protein families based on the presence of new sequence motifs without counterparts in public databases, while others containing known protein domains extend already existing families and also constitute new ones.

**Conclusions:**

We demonstrate that our multistep assembly approach allows *de novo *assembly of the newt transcriptome with an annotation grade comparable to well characterized organisms. Our data provide the groundwork for mechanistic experiments to answer the question whether urodeles utilize proprietary sets of genes for tissue regeneration.

## Background

The regenerative potential of urodele amphibians and especially newts as adult individuals has been known for more than 200 years. The complete regeneration of entire appendages [1] is one of the landmark abilities of newts accompanied by their ability to regenerate parts of the central nervous system [2,3], the lens [4] and the heart (reviewed in [5,6]). Compared to other animal models [7,8] the potential of the adult red spotted newt for regeneration is remarkable. Newts do not lose the capacity to regenerate the lens even after repetitive tissue damage that continues over several years. Lenses remain indistinguishable in their molecular signature and morphology even after repetitive rounds of regeneration [9]. In sharp contrast, the ability of mammalian species to regenerate declines rapidly during postnatal life, suggesting that the regenerative capacity in mammalians is inversely proportional to the age of an individual. At present, it is still unclear whether regeneration in mammals is a mere extension of embryonic development or represents an independent process. It seems likely that a thorough analysis of the molecular mechanisms of newt tissue regeneration will aid our understanding of regenerative processes and help to develop new therapeutic strategies.

Although the regenerative capability of the newt is extraordinary, it has attracted less attention than other model organisms in recent decades. This is partly due to the comparatively long reproductive cycle of newts and their enormous genome size, estimated to reach c × 10^10 ^bases, which is about 10-times the size of the human genome. Therefore, no genome sequencing approach has so far been initiated and only about 140 annotated transcript and protein sequences are available in public databases (NCBI, as of September 2011). To overcome these obstacles, several initiatives were launched to obtain more detailed 'omics' data. A set of 11,000 EST sequences [10] was uploaded to public databases and a mass spectrometry-driven proteomics approach was able to identify peptides for more than 1,000 newt proteins [11]. Furthermore, we devised a comprehensive newt data depository providing the ability to store, retrieve, link and visualize sequences, proteins and expression data [12]. This repository allows implementation of comprehensive datasets derived from next generation sequencing experiments and high-throughput proteomics.

Sequencing technologies have seen rapid progress in recent years with respect to the amount of base calls and price. Despite these advancements and dramatic price cuts, the large size of the newt genome still plagues *de novo *genome projects and makes them hardly affordable. An obvious solution to this problem is the analysis of transcriptomics data, but a detailed analysis of such data is difficult in the absence of a comprehensive reference dataset. The availability of a detailed reference transcriptome of the newt *Notophthalmus viridescens *would also yield functional insights and allow identification of new and known proteins that might be instrumental in tissue regeneration of urodelian amphibians.

Here, we present the *de novo *assembly of the newt transcriptome, based on hybrid sequencing datasets derived from Sanger, 454 Roche and Illumina platforms. Our approach, which generated over 38,000 unique transcripts with high quality annotations, covers embryonic and larval development, different stages of heart, appendage and lens regeneration and a comprehensive collection of tissue-specific transcripts. To exclude sequencing artifacts and verify coding sequences, transcriptome data were matched to a large mass spectrometry-derived proteomics dataset, resulting in the identification of 14,471 newt transcripts with approved protein-coding capacity. Further bioinformatical analysis disclosed several new protein families exclusive to urodelian amphibians, of which some contain known domains from public databases, but also entirely new clusters of proteins sharing sequence motifs not known in other species. We reason that some of the proprietary newt proteins might play important roles in regeneration processes unique to urodeles.

## Results

### Library construction and *de novo *assembly strategy

To achieve a broad coverage of the newt transcriptome, we used 48,537 EST clones of a normalized cDNA library derived from regenerating newt hearts (uninjured, sham, 2 h, 6 h, 12 h, 24 h, 48 h, 4 days, 7 days, 14 days, 21 days, and 35 days after mechanical cardiac damage) previously described in [12]. In addition, we generated 807,466 reads from a complex normalized cDNA library by pyrosequencing using a 454 platform with an average read length of 310 bp. The normalized library represented all stages of embryonic and larval development, different stages of heart, appendage and lens regeneration and a comprehensive collection of transcripts from multiple adult tissues (see Materials and methods). Finally, we produced a set of 679,816,626 Illumina paired end reads, (2 × 60 bp, insert size 150 bp), which were derived from a cDNA library of both dorsal and ventral iris during lens regeneration, 4 and 8 days post-lentectomy.

Next, we evaluated four different approaches to achieve an optimal assembly of different sequence reads, since there is no gold standard for the combination of sequences derived from different sequencing platforms. Our purpose was to enlarge the N50 (length N for which 50% of all bases in the assembly are located in a transcript of length L < N) and the total number of input sequences, the number of assembled transcripts over 1,000 bp and to decrease the number of sequences shorter than 400 bp. The N50 value was only used as a surrogate parameter since we are well aware that N50 values might be affected by the presence of a few, very long transcripts. Hence, we also tested effects of the choice of k-mers and the use of a reference mapping strategy, which are known to play crucial roles in the efficiency of an assembly [13].

The first approach was based on initial mapping of 454 and Illumina reads to preassembled Sanger reads (reference mapping) to reduce complexity and redundancy of the datasets. This strategy also enabled us to determine the extent of the new sequence information that was added by 454 and Illumina sequencing. 454 and Illumina reads that remained after mapping were used for individual *de novo *assemblies. Almost 90% of the remaining 454 reads were assembled using MIRA while the extent of assembled Illumina paired end reads using Velvet and Oases ranged from 20% to 40% depending on the k-mer choice. All resulting contigs from individual *de novo *assemblies and preassembled Sanger reads were pooled in a final assembly with 129,474 transcripts and a N50 of 776 bp.

For the second approach, we focused on the paired end information of short reads. We tested the influence of unpaired read mapping and subsequent scaffolding on the assembly by mapping all Illumina reads to preassembled Sanger reads (reference mapping) as in the first approach and by subsequent mapping to preassembled 454 contigs (reference mapping). Remaining reads were assembled *de novo *(Velvet and Oases) without considering paired end information. The significantly enlarged number of short contigs (373,288 in the n8dd pool to 696,587 in the n8dv pool) was scaffolded by SOPRA. All contigs from Sanger, 454 and scaffolded Illumina reads were then assembled by TGICL/CAP3. This second approach yielded a N50 of 753 bp, including 118,416 transcripts.

In the third approach we evaluated the influence of reference datasets on the outcome of the assembly. Since there is no reference genome or transcriptome available for the newt, we used an EST dataset from *Cynops pyrrhogaster*, which is closely related to *N. viridescence*. The EST dataset included 25,747 sequences with an average length of 830 bp. Again we mapped our short reads to preliminary assembled Sanger reads and the remaining reads to sequences from *C. pyrrhogaster*. Residual non-mapping reads were assembled *de novo *(Velvet and Oases) and all contigs (assembled 454 reads as well as reads mapped to *C. pyrrhogaster*, and *de novo *assembled Illumina reads) were assembled via TGICL/CAP3 followed by addition of preassembled sanger reads. The resulting assembly had a N50 of 801 bp including 151,118 transcripts.

**Figure 1:**
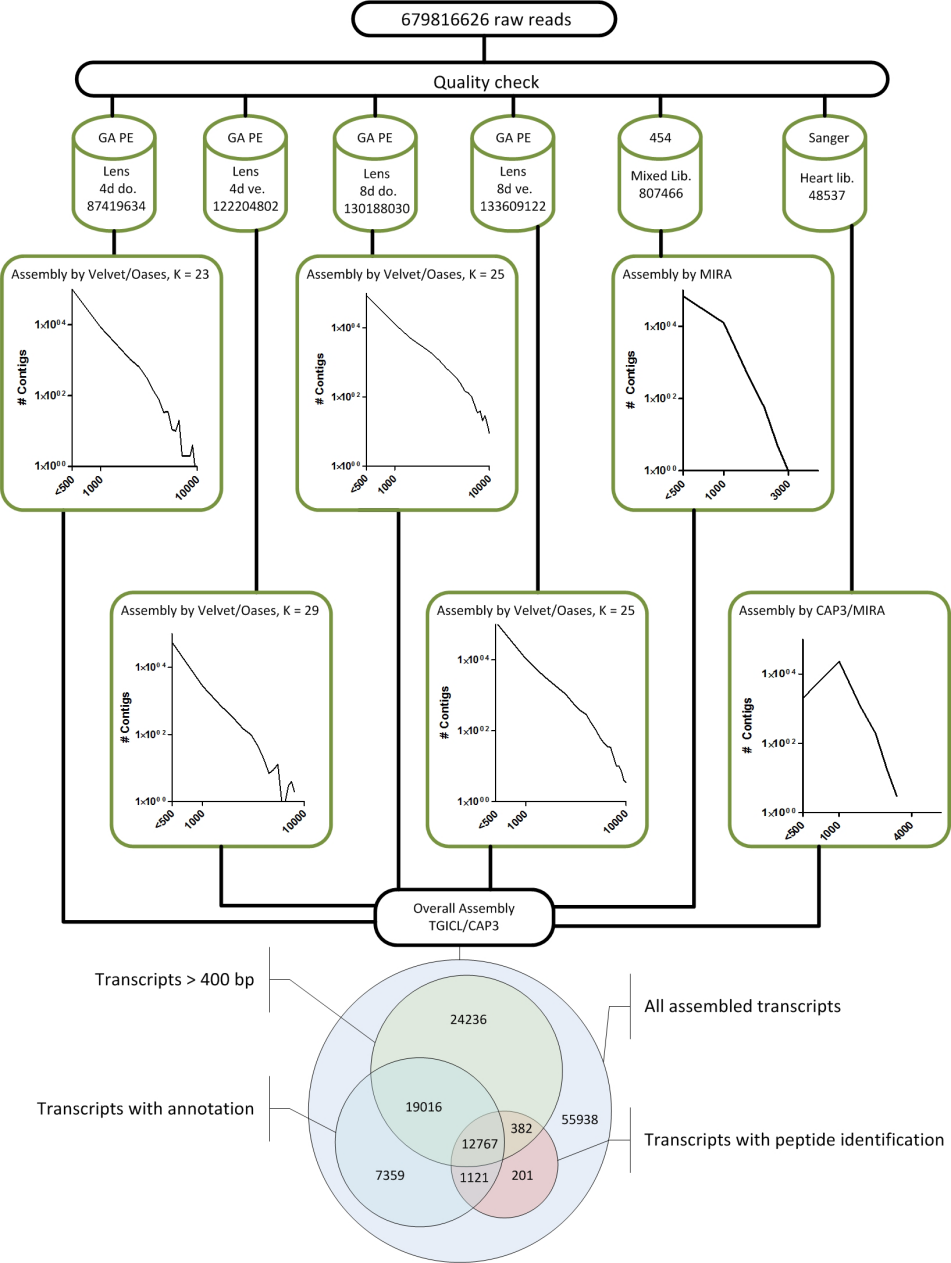
**Workflow of the *de novo *assembly and resulting annotation steps**. **(a) **Raw reads were filtered and assembled for each sequence pool. **(b) **Optimized assembly strategies for individual Illumina, 454 and Sanger sequence pools illustrated as number of contigs versus length distribution. Velvet and Oases were used for the Illumina reads, MIRA for the 454 reads and CAP3 and MIRA for Sanger reads. Contigs from individual assemblies were merged by TGICL and CAP3, yielding 120,922 unique transcripts **(c) **Venn diagram of all assembled transcripts, categorized into transcripts larger 400 bp, transcripts with annotation, and transcripts with peptide identification. GA, genome analyzer; PE, paired end.

Our final approach, which was eventually used for the generation of the final reference transcriptome, was based on a two-step strategy to reduce redundancies and to minimize computational time required for further analysis. In the first step, each sequence pool was assembled independently without a mapping step (Figure 1a). For the Illumina paired end reads, assembled by Velvet and Oases, we tested several k-mer parameters and continued with the best performing set-up (Figure 1b). The 454 sequence pool was assembled by MIRA, the Sanger sequence pool by CAP3 and MIRA (Figure 1b). In the second step, all resulting contigs irrespective of their length were used for a hybrid assembly performed by TGICL and CAP3 employing mgblast to remove redundancies. This strategy yielded 120,922 putative non-redundant transcripts with an N50 of 975 bp (Figure 1c). The last strategy yielded the highest N50, without a significant drop in the number of individual transcripts compared to our other approaches and hence was chosen for all further annotation and verification steps (Additional file 1).

### High quality annotation of the transcriptome

The final assembled transcriptome dataset contained 120,922 transcripts, which provided an approximately 20-fold higher number of non-redundant assembled transcripts compared to previous studies [10,12]. These transcripts were annotated by homology searches using the BLAST algorithms. To identify transcripts with a substantial similarity to known sequences, we set the e-value cutoff to e-15, although the total number of annotated sequences with a reasonable similarity dropped significantly compared to higher e-value cutoffs. At least one hit classified as 'homology verified' was detected for 38,384 individual transcripts. To determine similarities to sequences with known protein coding potential, we performed searches against protein and nucleotide databases (NCBI nr and nt databases). To disclose additional similarities to sequences from organisms that are not included in the above-mentioned databases, such as other urodele amphibians, we also performed searches against EST databases (NCBI EST human, EST mouse and EST others).

To enable a preliminary functional analysis we needed high quality identifiers for identified transcripts. Therefore, we performed searches in Uniprot databases for the species mouse, human and cow, which show good quality of annotation. Additionally, we used Uniprot databases for zebrafish, *Xenopus *and chicken. The zebrafish served as another model organism for tissue regeneration, whereas *Xenopus *and chicken are the closest relatives to newts in the evolutionary tree with a substantial number of Gene Ontology (GO) annotated proteins. For these searches, the e-value threshold was set to e-10 since many entries in the Uniprot database are manually curated. We generated functional annotations for 30,760 transcripts, including all searched species. Taken together, we annotated around 40% of our complete *de novo *assembled transcript pool (not filtered for sequence length).

**Figure 2:**
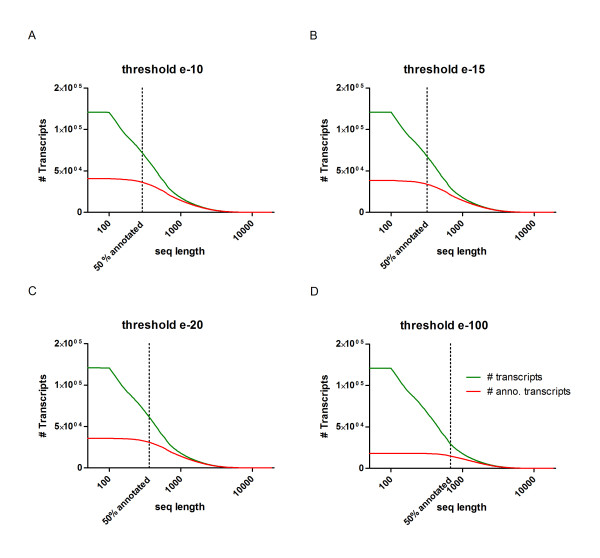
**Influence of e-value cutoff on transcript annotation rate**. **(a-d) **Number of annotated transcripts (y-axis, log scale) by cut-off e-value plotted against sequence length (x-axis log scale). The total number of transcripts is shown in green, and the subset of annotated transcripts is shown in red. The number of transcripts with an annotation increases with the length of transcripts. The percentage of annotated transcripts depends on the cutoff e-value threshold. For example, an annotation of 50% of transcripts at 290 bp is reached at a cutoff of e-10 (a), this length increases to 680 bp when a cutoff of e-100 is used (d). We selected the minimum sequence length of 400 bp for our assembly to achieve a sufficient annotation rate of assembled sequences.

Furthermore, we evaluated the effect of transcript length and e-value cutoff on the rate of sequence annotation (Additional file 2). Not surprisingly, we found that sequence length filtering improved annotation rates considerably. Length filtering also helped to distinguish roughly between short non-coding sequences and sequences with coding potential. We first sorted all transcripts by sequence length and grouped them by bars of 50 bp that were plotted relative to their frequency (Figure 2). In the same graph, we plotted the subset of annotated transcripts relative to sequence length. The rate of 50% annotated transcripts was reached at a sequence length of 290 bp using an e-value threshold of e-10 (Figure 2a; including annotations in NCBI and Ensemble databases). The sequence length corresponding to 50% annotation rate depended on the e-value threshold and was continuously increasing to 320 bp for e-15, 360 bp for e-20 and 680 bp for e-100 (Figure 2b-d). Based on these findings, we defined a sequence length filter of 400 bp to distinguish between primarily coding sequences and sequences of mostly unknown function. The annotation rate for sequence length ≥400 bp with a threshold of e-15 was 56%, which corresponds to 56,401 remaining transcripts. This shows that the number of annotated transcripts is enriched for longer transcripts.

### Bioinformatical assessment of the coverage of the newly established newt transcriptome

The main purpose of the generation of a reference newt transcriptome was to establish a comprehensive resource for future next-generation sequencing and high-throughput proteomics approaches. Hence, our transcriptome assembly and further validation by high-throughput protein analysis favored high quality annotations and not inclusion of maximum numbers of transcripts and proteins. The coverage of our reference transcriptome was estimated by determining the coverage of general signal transduction pathways and the rate of identified members of known gene families. We analyzed more than 850 signal transduction pathways listed in BioCarta (San Diego, CA, USA), KEGG [14], Reactome [15] and other databases and determined the rate of coverage using our transcripts with high quality annotations from Uniprot databases. For example, we covered over 80% of the human p53 signaling pathway (58 components) listed in KEGG (Additional file 3). Similarly, our approach identified more than 80% of all transforming growth factor beta signaling pathway members (Additional file 4). Similar numbers were reached for other pathways analyzed. The lack of complete coverage might be due to the stringent cut-off criteria used for high quality annotations and/or might be caused by interspecies differences in the number and composition of signal transduction pathways. Further sequencing projects and refined bioinformatical analysis might solve this conundrum in the future.

To further investigate the fragmentation grade of the transcriptome in the absence of comprehensive genome data, we assigned orthologues by a recursive best mapping step to all taxa represented in the Uniprot database. We were able to identify 3,771 ortholog pairs by sequence similarity and investigated the ortholog pair alignment length. We found newt transcripts that were between approximately 5% and 25% longer than the corresponding orthologue sequences. Based on the identified length variation (25%) we assumed that all newt transcripts with alignments >75% of orthologue sequences are full length, yielding 2,000 (53%) 'full length' candidates. The complete list of candidates is presented in Additional file 5.

### Experimental validation of the protein coding potential of *de novo *assembled transcripts by high-throughput mass spectrometry

To validate the coding potential of the newly established newt transcriptome, we performed numerous mass spectrometry (reverse-phase nano-liquid chromatography coupled to a tandem mass spectrometry (LC-MS/MS)) experiments and also used mass spectrometry data from earlier studies. Proteins were isolated from various newt tissues, including heart [16], lens, tail [11], liver and spleen at different time points during regeneration and from uninjured tissues. Additionally, we isolated proteins from the newt-derived myoblast-like cell line A1 [17] during different stages of differentiation into myotubes. Stable isotope labeling by amino acids in cell culture (SILAC) of newt tissues enabled us to filter for mass shifted spectra, which increased the quality of the dataset considerably [11]. Peptides identified by LC-MS/MS measurements were compared to a protein database generated by reverse translation of all potential coding sequences of the newt transcriptome using all possible reading frames. We identified 55,605 different peptides that matched to 14,471 different transcripts, which corresponds to 11.97% of the total number of 120,922 non-redundant transcripts. In total, 11,113 transcripts had at least two peptides. These numbers correspond well to results from similar studies and reflect the lower sensitivity of mass spectrometry-based protein detection methods compared to nucleotide sequencing approaches [18]. The transcript with the highest number of peptides (266 peptides, 17,373 nucleotides) coded for plectin, an approximately 4,500 residue protein. This protein included five frame shifts. In total, 3,618 transcripts carried a frame shift as identified by peptide assignments. See Additional file 6 for an example of the identification of ORF shifts.

**Figure 3:**
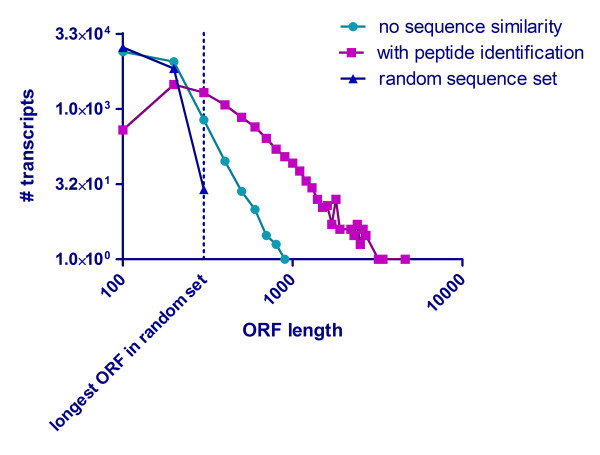
**Estimation of coding potential in non-annotated transcripts**. ORF coding potential in a randomly generated transcript set, verified transcripts without sequence annotation, and sequences with high quality annotations. All three transcript sets containing sequences longer than 400 bp were translated in six reading frames. The longest ORF per transcript was plotted against the number of transcripts per dataset. Note that a significant number of ORFs from the dataset without annotation (cyan line) exceeds the maximum ORF length of the randomly generated transcript. The non-annotated group contains an identical number of total transcripts with identical sequence length (blue line), indicating the existence of potential newt proteins that were not identified by proteomics.

Interestingly, we were unable to find any similarity for a substantial number of assembled sequences >400 bp to other transcripts or proteins. To estimate the coding potential, all transcripts were translated in six ORFs. The longest ORF per transcript was plotted as a function of frequency and compared (i) to a randomly generated dataset containing transcripts of the same number and length and (ii) with transcripts with proven coding potential based on matching peptides identified by mass spectrometry. A substantial number of transcripts from the dataset containing sequences with no similarity exceeded the maximum coding potential of transcripts from the randomly generated control set (Figure 3). We therefore concluded that the newt genome contains a large number of proprietary protein coding genes with limited similarity to known genes from other organisms. In the future, additional proteomics experiments using isolated cells and subcellular fractions together with the continuous increase of sensitivity and dynamic range of mass spectrometry instruments might allow detection of lower abundant proteins from newt tissues, thereby enabling identification of more unknown urodelian-specific proteins.

### Identification of new urodelian-specific proteins and protein families

**Figure 4:**
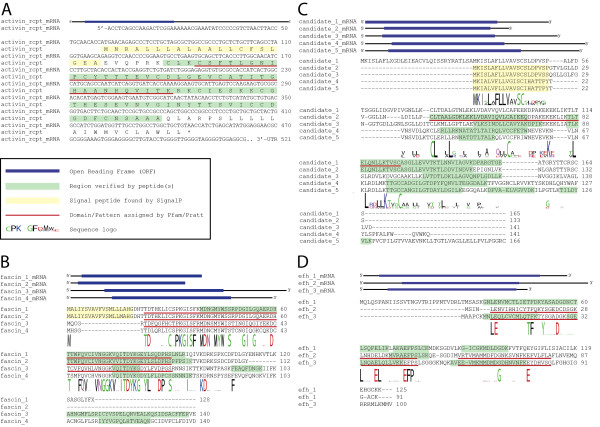
**Identification of new urodele-specific protein families**. Schematic transcript sequence indicates flanking UTRs and included ORF with start and stop codon marked in blue. Amino acid regions with peptide coverage are marked in green, significant signal peptides are marked in yellow. Detected domains or patterns are highlighted by a red underline. Within each multiple sequence alignment, a logo represents sequence conservation. **(a) **New protein including an activin receptor domain. **(b) **Protein cluster of four members detected by a common fascin domain. **(c) **Sequence set representing a group of similar proteins detected by a common pattern, missing any known domain. **(d) **Combined sequence cluster, including one member with Pfam domains S100 and EF-hand like and three members without these domains.

We next wanted to characterize transcripts that most likely encode new proteins not present in non-urodelian species. Such proteins might be involved in biological processes characteristic for newts, such as tissue regeneration, or reflect other species-specific properties. We focused on transcripts that either lacked similarities to any entries in public databases or showed sequence similarity exclusively to those from other urodelian amphibians. To avoid sequencing artifacts we filtered for sequences that encode peptides measured by mass spectrometry and hence represent valid protein coding genes. We identified 583 protein-coding transcripts that did not show any hit in public databases and 243 protein coding transcripts with similarity to urodeles only (Figure 1c). Next, we screened the resulting 826 sequences for conserved domains or motifs using the Pfam database [19] to facilitate assignment of putative protein functions. We identified 145 defined domains within 131 transcripts (Additional file 7) while the remaining 695 transcripts did not contain any known motif. Domains located in urodelian-specific transcripts covered a wide spectrum of known domains, including an activin receptor domain (Figure 4a), which is also present in human mutant transforming growth factor receptor beta receptor I fragment (A6MIV6_HUMAN in the Pfam database). Other examples included the fascin domain (Figure 4b), which characterizes a family of structurally unique actin cross-linking proteins. The diversity of identified motifs suggests that various biological decisions are influenced by urodelian-specific proteins, although a precise delineation of potentially affected processes is not possible at present.

**Figure 5:**
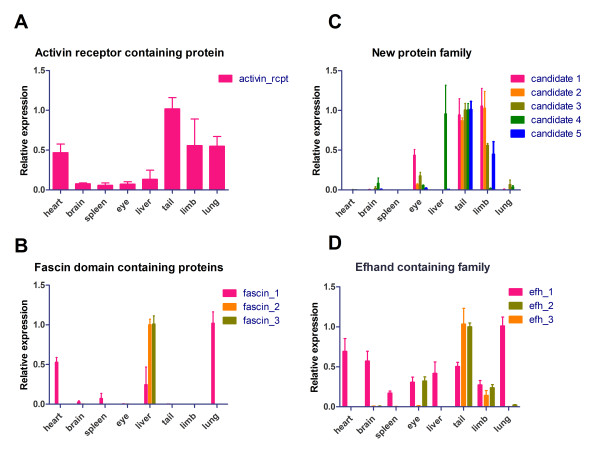
**Expression of newly identified genes in adult tissues of the newt**. Real time RT-PCR analysis (n ≥ 3) of selected candidates and families in adult heart, brain, spleen, total eye, liver, tail, limb and lung. Tissues with the highest expression level for each candidate were normalized to a relative value of 1.0.

To further investigate the tissue-specific distribution of some of these urodelian-specific transcripts, we performed RT-PCR using a set of tissues, including the heart, brain, spleen, eye, liver, tail, limb and lung. We found an increased expression of the activin receptor domain containing protein in the tail and a moderate expression in the heart, limb and lung (Figure 5a). A more basal expression was detected in the remaining tissues, which suggests that this protein is expressed in muscle-containing tissues. To study whether the activin receptor domain containing gene responds to regenerative processes, we analyzed changes in expression during regeneration of the lens and heart by cDNA microarrays. The heart arrays are accessible via the newt repository [12]. A detailed analysis of lens arrays will be published elsewhere [20]. A combined overview of heart and lens arrays is provided in Additional files 8 and 9. We detected a uniform expression in the regenerating heart tissue, and a slight up-regulation in the dorsal and ventral iris (Additional file 10). Further validation by quantitative RT-PCR revealed a significant up-regulation of the activin receptor domain containing gene in the regenerating heart 21 hours after injury and in the dorsal iris 3 days after lentectomy (Additional file 11a). Furthermore, we found that two members of the fascin domain containing protein family were exclusively expressed in the liver whereas another family member was highly expressed in the heart and the lung but only barely detectable in the liver (Figure 5b). Interestingly, we saw a strong expression of the heart/lung fascin domain containing gene during regeneration of the heart and lens with a strong up-regulation 2 days after cardiac injury, which persisted until 35 days after injury with an expression peak at approximately 14 days after injury (average ratio 2.7). This pattern was also corroborated by quantitative RT-PCR analysis (Additional file 11b). A similar trend was observed in dorsal iris microarrays where the expression was increased 5 days after lentectomy (average ratio 1.3). The ventral iris showed no up-regulation at any time point (Additional file 10). Since lens regeneration originates from the dorsal iris but not from ventral side, we would like to speculate that this fascin domain containing gene is involved in initiation of the regenerative process [21].

Next we tried to resolve more protein clusters in the remaining 695 proteins that do not contain any known domains or motifs. Therefore, we performed PSI-blast searches (five iterations, cutoff 0.05). After manual inspection, we selected several clusters for further investigation. Finally, we used the PRATT tool [22] to scan all sequences as well as manually selected clusters identified by PSI-blast for new patterns not present in public databases such as PROSITE [23]. Using this approach we identified several clusters, which are likely to represent new protein families. One of these clusters consisted of five members represented by complete ORFs and additional 5'/3' UTRs (Figure 4c). All members of this cluster shared a common signal peptide, which indicates that these proteins are secreted [24]. The family is defined by the common motif (L-x(1,3)-C-L-x(2)-[AL]-L-x(3)-[AL]-[AET]-x(2)-[LV]-x-[AS]-[ILV]-x-[DQ]-[LV]-[LV]-C-[AC]-[FIV]-x(3)-[DN]-[EP]-[AIV]-[EK]-x-K-[EN]-x-L). Each sequence was covered by at least two peptides measured by mass spectrometry. All five family members lacked any similarity to known sequences from other urodeles. However, due to the limited sequence information available, it is difficult to exclude that related protein families do also exist in other urodeles. RT-PCR-based expression analysis disclosed that all genes of this newly defined group were highly expressed in the tail, four were highly expressed in the limb and one was strongly transcribed in the liver but not in the limb (Figure 5c). Additionally, one gene (candidate 1) showed a moderate expression in the eye. No member of this gene family yielded significant signals on our heart and lens microarrays. Quantitative RT-PCR analysis during lens regeneration revealed significant expression changes of newly identified candidate genes 2 to 5. Candidate gene 2 was significantly down-regulated in the dorsal but not the ventral iris after lentectomy. Candidate gene 3 was also significantly down-regulated in the dorsal iris with a similar trend in the ventral iris. In contrast, candidate 4 was significantly up-regulated in the dorsal iris during regeneration with a peak 3 days after lentectomy. Lastly, candidate gene 5 showed a highly interesting, inverse expression pattern in the dorsal and ventral iris (Additional file 11c).

Finally we performed a combined PSIblast and PRATT analysis including all 826 sequences to identify protein clusters consisting of members with and without known domains. From 380 sequences that clustered with at least one other sequence, we identified additional protein clusters of which one example is a set of three sequences (Figure 4d). Two members were characterized by the presence of S100 and EF-hand domains identified by Pfam search, but did not show any similarity to urodele EST sequences, while the additional member, which was mapped to the initial sequence by PSI Blast, showed similarities to known ESTs from other urodelian species. All transcripts contained a complete ORF flanked by 5' and 3' UTRs. Sequence similarities within this family were visualized by multiple sequence alignment and display of sequence logos [25]. Our mass spectrometric measurements covered all family members with several peptides, thereby corroborating the existence of the respective proteins. Individual members of this family showed distinct expression patterns (Figure 5d). The first family member (efh_1) was present in virtually all tissues tested with strongest expression in lung and heart. The second family member showed strong expression in the tail and limb and moderate expression in the eye (efh_2). The third family member (efh_3) was only present in the tail and limb tissue, suggesting a skeletal muscle-specific expression pattern. Based on our microarray expression, we found a slight up-regulation of the efh_1 member in the heart 21 days after injury (average ratio 0.6) and a down-regulation in the ventral iris (average ratio -1.7). A more detailed expression analysis of EF-hand family members during heart, limb and lens regeneration revealed a slight up-regulation of Efh1 in regenerating limbs starting from 0 days to 14 days and a strong increase in the dorsal iris between 1 day and 3 days (Additional file 11d). Efh2 was down-regulated in the ventral iris but strongly up-regulated at 3 days in the dorsal iris before its expression decreased during the course of regeneration (Additional file 11d). Finally, Efh3 was significantly up-regulated at 14 days in the regenerating limb, which was mirrored by increased expression in the dorsal iris 1 day after injury. In contrast, Efh3 was dramatically down-regulated after injury in the ventral iris, which does not contribute to the regenerating lens (Additional file 11d). Nucleotide and amino acid sequences of candidate molecules are given in Additional file 12.

## Discussion

High-throughput sequencing has become an indispensable tool for whole-genome analysis of complex organisms, a trend that has also been fuelled by decreasing sequencing costs. Nevertheless, *de novo *whole-genome analysis is still costly and needs specific bioinformatical expertise and dedicated computational equipment, especially for organisms with very large genomes. Alternatively, *de novo *whole-transcription analysis represents an attractive option to gain detailed insights into the genetic constitution of an organism at a fraction of the costs and efforts needed for whole-genome analysis. In fact, this approach has made several organisms amenable for molecular analysis that have not been the subject of genome projects despite the valuable information that might be gained by comprehensive genetic analysis. Examples include exotic insects [26], crustaceans [27], planarians [28] and several more. Transcriptomes of such organisms were decoded by mapping transcripts to a reference genome of phylogenetic close relatives or by *de novo *assembly of transcripts. Assembled transcriptomes are used as reference sets for RNA expression analysis, as matrices for mass spectrometric-driven peptide/protein identifications or for phylogenetic analyses. The availability of annotated transcriptomes also enables RNAseq analyses of niche organisms, which bypasses previous limitations due to the lack of high-density microarrays.

The red spotted newt, *N. viridescens*, is an organism with remarkable tissue regeneration abilities. Although the regenerative potential of this animal has been known since 1712 [29-31] and was intensively investigated from the 1950s to the 1980s [32-39], no genome or larger sequencing project on it has been initiated so far, which is mainly due to the very large genome size (approximately c × 10^10 ^bp) of newts. Here we describe the first comprehensive *de novo *assembled transcriptome together with a large-scale experimental validation of coding sequences.

Since the content and quality of a *de novo *assembled transcriptome strongly depends on the input material, choice of sequencing platforms and bioinformatical processing, we devised an integrated strategy to achieve the best possible outcome. First, we used complex RNA pools covering embryonic and larval development, the entire process of heart, appendage and lens regeneration and a comprehensive collection of tissue-specific transcripts to limit the problem of incomplete representation of mRNAs. Furthermore, cDNA libraries were normalized to increase the probability to detect mRNAs expressed at low levels or in a small number of cells. Second, we combined the output from different sequencing platforms, which each have specific advantages: long reads with high accuracy were obtained by Sanger sequencing; medium read lengths from normalized cDNA libraries at medium depth were generated by the 454 platform; and short paired end reads with high sequencing depth were generated from the Illumina platform. Third, we tested five different assembly strategies in combination with several assembler software packages since most *de novo *assembly tools are not suited to process data from different platforms and the choice of adjustable parameters of the bioinformatics tool has a significant impact on the success of a *de novo *assembly. Our assembly strategy, which is based on preassembly of single sequencing sets using custom-configured assemblers followed by subsequent hybrid assembly into one transcriptome, outperformed a 'reference mapping' like approach based on sequences available for a close relative of the newt (*C. pyrrhogaster*). We obtained larger N50 values than in reference mapping approaches without a significant reduction in the overall number of unique transcripts.

The length, distribution and number of *de novo *generated transcripts give only a rough estimate about the quality of a *de novo *transcriptome assembly. It is crucial to accomplish a comprehensive annotation of all transcripts and validate putative coding RNAs. To achieve this goal we used multiple BLAST algorithms and fine-tuned e-value thresholds, allowing detection of protein coding transcripts with different degrees of evolutionary conservation. The annotation rate of 56% that we achieved is similar to other published transcriptome assemblies (*Schmidtea mediterranea*, 6,729 annotated from 18,619 transcripts [28]), although we could not rely on comprehensive annotation of closely related organisms. Furthermore, the good coverage (around 80%) of components of known signal transduction pathways indicates that we identified the majority of protein coding transcripts that exist in *N. viridescens*. Nevertheless, we would like to point out that we might have missed transcripts or proteins that are only lowly expressed in the tissues studied, which might compromise our goal of achieving a comprehensive list of newt protein coding genes to a certain extent. In this context it is interesting to note that even well annotated model organisms such as zebrafish and mouse still carry a large number of non-annotated transcripts. For example, 40% of all transcripts present on the Affymetrix Mouse 430-2 array have either no gene symbol (5,553 probe sets), or only automatically assigned symbols such Riken (3,494 probe sets), predicted (506 probe sets), hypothetical (322 probe sets), expressed (505 probe sets), cDNA (175 probe sets) and families with similarity (230 probe sets). The Affymetrix Zebrafish Genome Array shows an even higher rate of almost 45% weakly annotated transcripts. From 15,122 entries, 2,773 lacked any gene symbol and 3,972 had only automatically assigned symbols (wu:fa, si:zfos, sb:cb and others). Expression analysis of a selected subset of newly identified genes by RT-PCR and microarray analysis revealed tissue-specific expression patterns as a pronounced response to heart and lens regeneration. A more thorough analysis of newly annotated transcripts during tissue renewal will help to define the regulatory network of genes controlling regeneration.

Taken together, our assembly and annotation strategy yielded annotated transcripts that are close to organisms with complete genome information, although the focus of our approach was to obtain transcripts with high quality annotation rather than a maximum number of transcripts. We do not claim that we have assembled and annotated the complete transcriptome since no sufficient sequence similarity fulfilling our significance criteria (e-15) was found for 44% of all assembled sequences (>400 bp) but our dataset is sufficient to serve as a matrix for high-resolution expression analyses.

It seems reasonable to assume that a significant part of the non-annotated transcripts represent artifacts generated by misassemblies. Alternatively, such sequences might also represent non-coding RNAs or mRNAs coding for newt- or urodele-specific proteins. However, we did only find a limited number of non-coding RNAs. Screening for non-coding RNAs using the noncode database [40] yielded 17 and 24 hits with high sequence similarity when transcripts <400 bp and >400 bp were used, respectively. This lead us to the conclusion that many of our non-annotated sequences represent weakly conserved 5'/3' UTRs or that the newt has a large number of RNAs with yet unknown function and similarity to other organisms. Since the lack of information about newt proteins makes it difficult to distinguish between these possibilities, we took advantage of a large set of proteomics data from newts obtained by mass spectrometry. This approach not only enabled us to validate almost 15,000 transcripts as protein coding but also enabled the identification of 826 urodelian-specific proteins, which showed either no sequence similarity at all (583 transcripts) or similarities to urodele EST sequences only (243 transcripts). We would like to emphasize that these numbers most likely represent only the tip of the iceberg since the current sensitivity and dynamic range of mass spectrometry only allows measurement of high abundance proteins. Some of the validated new proteins belong in new protein families, while others contain defined protein motifs (15%) or no discernable primary sequence feature. The detection of new protein families was particularly intriguing and might indicate that founding members of the newly discovered families evolved further during urodele evolution to cope with species-specific requirements. In fact, it has been postulated that urodeles acquired regenerative capacity at the time when ancestral salamanders separated from the vertebrate tree [41], although more authors prefer the hypothesis that regeneration is a primordial property of metazoa [42], which was lost in most tetrapod vertebrates during evolution but selectively maintained in salamanders. Interestingly, previous studies identified newt-specific genes such as *Prod1*, a critical determinant of proximodistal identity in the limb bud, that mediates nerve-dependent signals to the regenerating blastema by utilizing a conserved signaling machinery [43]. Another example is nsCCN, a newt specific member of the CCN protein family that is exclusively expressed in regenerating but not in uninjured hearts [44]. Further functional studies will reveal whether the newly discovered families are drivers of regenerative processes or serve another, yet unknown cause. Of course, our conclusions are based on currently available sequence data, leaving the possibility that the newly discovered urodelian-specific proteins exist also in other species that have not been analyzed so far. The newly established *de novo *transcriptome of *N. viridescens *will be an indispensable resource for better understanding regenerative events in newts and facilitate the identification of molecules, conserved or urodele-specific, that control this fascinating process. We would also like to suggest that the combined use of transcriptomics and proteomics approaches provides a powerful means to address new model organisms and detect new protein coding genes.

## Conclusions

Despite several obstacles to manipulate and analyze the red spotted newt, *N. viridescens*, at the molecular level, its remarkable tissue regeneration abilities define this organism as an excellent model to study regeneration. The rapidly evolving techniques for genome/proteome analysis and genetic manipulation will help to understand regenerative processes at a functional level. Here, we describe the first comprehensive *de novo *assembled transcriptome of *N. viridescens *combined with large-scale experimental validation of coding sequences. The use of complex RNA pools and normalized cDNA libraries allowed us to cover different biological processes, including embryonic development, as well as heart, appendage and lens regeneration. *De novo *assembly of the newt transcriptome using different computational strategies was facilitated by combination of the output from different sequencing platforms, which each have specific advantages. The resulting 56% annotation rate of the transcriptome is similar to other transcriptome assemblies (*S. mediterranea*, 6,729 annotated from 18,619 transcripts [28]), which are less challenging compared to the newt. Finally, integration of transcriptomics and proteomics data allowed us to confirm the protein coding potential of almost 15,000 transcripts, resulting in the identification of 826 urodelian-specific proteins. Several of these newly identified proteins represent new members of defined protein families or completely new protein families and show distinct expression profiles during regeneration. The newly established transcriptome of *N. viridescens *provides a matrix for high-resolution expression analyses and will be an indispensable resource for a better understanding of regenerative processes in newts at the molecular level.

## Materials and methods

### 454 sequencing

Total RNA from regenerating heart (11 timepoints, 2 sham timepoints + undamaged), regenerating limb and tail (6 timepoints + undamaged), brain, eye, liver, lung, spleen, kidney, testes, ovaries, 1 cell embryo to larval stage 46 was extracted with Trizol (Invitrogen, Carlsbad, CA, USA) following the instructions of the manufacturer. Double-stranded cDNA was synthetized with the MINT Kit (Evrogen, Moscow, Russia) and cDNA was normalized with the Trimmer Kit (Evrogen). Library preparation for sequencing was done according to the GS FLX Titanium protocol provided by the manufacturer.

### Illumina paired-end-sequencing

Total RNA was extracted from the dorsal and ventral part of the iris 4 and 8 days after lens removal. Ribosomal RNA was depleted using the RiboMinus Eukaryote Kit (Invitrogen). Depleted RNA (1 µg) was processed according to the Illumina mRNA sample preparation guide. A-tailed DNA was ligated with paired end adaptors using T4-DNA ligase provided by the Illumina RNA-seq kit (Illumina, San Diego, CA, USA). Size selection of adaptor ligated DNA was performed by cutting the target fragment (400 to 450 bp) from the DNA gel. Amplification of the cDNA library was obtained by in-gel PCR. Cluster generation and sequencing were performed according to the cluster generation and sequencing manual from Illumina (Cluster Station User Guide and Genome Analyzer Operations Guide). Sequencing was performed by Cofactor Genomics (St Louis, MO, USA).

### Sanger sequencing

Total RNA from regenerating heart (11 timepoints, 2 sham timepoints + undamaged) was extracted with Trizol (Invitrogen). RNA was reverse transcribed to double-stranded cDNA with the SMART method and cDNAs were normalized by the DSN method (Evrogen). After cloning of cDNAs into pDNR-Lib vector, two independent bacterial libraries with more than 100,000 individual clones each were generated. After plating, 100,000 individual bacterial clones were picked and amplified in 96-well plates overnight. cDNA inserts were amplified by colony PCR. Products of PCR reactions were visually inspected on ethidium bromide stained gels and repeated if they had failed. Bacterial cultures were cryostocked in two replicates into 384-well plates. PCR products were precipitated, washed and resuspended at 200 ng/µl in an appropriate spotting buffer (3 × SSC, 1.5 M Betain) and again checked by visual inspection on ethidium bromide gels. We spotted 100,000 cDNA amplicons, including controls, onto two sets of glass microarrays (Nexterion slide E, Schott). After microarray hybridization, all spots showing a significant deregulation together with other robustly detected array spots were selected for Sanger sequencing. Around 52,000 individual colonies were selected for re-amplification and sequencing, yielding around 48,500 Sanger sequences of high quality. All individual sanger sequences are available via the Newtomics repository [12,45].

### Quality control of sequences and *de novo *assembly

Base calling for Sanger reads (48,537) was performed by Phred. Primary clustering was done by wcd [46], assembly by cap3 [47]. The *de novo *assembly yielded 26,594 unique transcripts. Illumina raw read quality was determined using FastQC quality control tool [48].

Illumina sequencing reads below a read quality threshold of 20 were trimmed base by base from the 3' end until the average quality of the read was >20. Paired end sequences having one read with a length less than 35 were discarded before assembly (Additional file 1). *De novo *assembly of Illumina sequences was performed on each library separately using Velvet [49] and Oases [50]. To choose optimal parameters we evaluated summary statistics like N50, number of contigs and percentage reads assembled for k-mers 23, 25, 29 and 35. The Oases tool was run with a minimum transcript length of 100 bp, insert length of 150 bp and minimum coverage of 5. 454 sequences were *de novo *assembled by MIRA [51] after quality check and adapter clipping, using the following parameters: job=denovo,est,accurate,454 -fastq -notraceinfo -noclipping -AS:sep=yes:urd=no 454_SETTINGS -AS:mrl = 100 -OUT:sssip=yes.

### Generation of unique transcripts

To lower the redundancy resulting from individual assemblies, all Illumina transcripts from Oases, Sanger transcripts from CAP3 and 454 transcripts from MIRA were pooled in one file. Using the TGICL/CAP3 pipeline [52] pooled transcripts were compared to themselves, using the mgblast (modified version of megablast [53]) algorithm. Clusters were generated with at least 90% sequence identity and a maximum unmatched overhang of 30 bp. For each cluster, all subcluster assembly results, that is, the biggest transcripts that are not contained in other sequences, were pooled with singletons and assembled a second time with CAP3. Resulting contigs and singletons from each cluster were stored in a globalContig and globalSinglets file. Remaining clusters with less than 15 transcripts per cluster were assembled together with CAP3 and added to a globalContigs or a globalSinglets file. Concatenation of these two files yielded non-redundant and unknown transcripts of five libraries.

### Sequence annotation and functional assignments

To annotate sequences obtained by *de novo *assembly, we performed sequence similarity searches using the BLAST algorithm. We implemented an automated annotation and quality filter pipeline, using the NCBI BLASTcl3 tool and UNIX shell scripts. The scripts performed blastn, blastx and tblastx searches on NCBI's nucleotide (nt), EST (human, mouse, other), protein (nr) and high-throughput genome sequencing databases. For the tblastx search of the NT database we used a high performance computing cluster and pipeline hosted at the GWDG (Gesellschaft für wissenschaftliche Datenverarbeitung mbH Göttingen). We set the e-value threshold to e-15. We performed a quality rating by checking for keywords, which are represented in weak description lines (like 'mRNA', 'cDNA', 'clone' or 'genomic'). Detected sequence similarities containing one or more of such keywords were marked as low quality hits. We collected at least three top hits per taxon, BLAST algorithm and database. We performed these quality checks for 90 taxa in total. Data are accessible via the Newtomics repository [45].

To assign functional annotations to *de novo *assembled transcripts, BLAST searches against GO annotated Uniprot databases (e-value threshold <e-20) from mouse, human, zebrafish, chicken and cow were performed to cope with the limited GO assignments for amphibians [54]. To avoid redundant functional assignments, the best-rated similarity hit with at least one GO annotation per taxon was chosen.

### Peptide identification

Protein samples were isolated from newt heart, lens, tail, liver and spleen at different time points during regeneration or from uninjured tissues. Additionally, proteins from a newt-derived myoblast like cell line (A1) during different stages of differentiation into myotubes were processed for mass spectrometry (reverse-phase nano-LC-MS/MS) measurements. Partially, tissues were labeled by SILAC *in vivo *as described [55]. Heavy amino acid derivatives of lysine (Lys6 and Lys8) and arginine (Arg10) were used for metabolic labeling. Analysis of individual mass spectrometry measurements is described elsewhere [55]. To identify mass spectrometry spectra, *de novo *assembled transcripts were translated into six reading frames to generate an Andromeda search engine compatible database [56]. Only reading frames longer than 25 amino acids were used for further analysis. The maximum false discovery rate was set below 1% for peptide and protein identifications using the DECOY target database approach [57]. For search transmission to Andromeda and peptide clustering, the MaxQuant software package (Version 1.2.0.18) was used [58].

### Analysis of unknown, peptide verified sequences

Pfam [19] batch sequence search was used with default parameters to identify known domains within the subset of 826 coding transcripts without similarities to entries in public databases. Results were inspected manually. Detection of protein clusters sharing motifs or domains was done using PSI-Blast. For each sequence, a Blast database was generated from 825 protein sequences excluding the query sequence. Five iterations of PSI-Blast searches were used with a cutoff value of 0.05. Resulting clusters with at least two sequences were inspected manually to find candidates for new motifs or domains. PRATT [22] was used with parameters -c% 0.6 -FL 4 -FN 3 -PX 2 to search for common patterns within all protein sequences to generate clusters of size 6 and maximizing fitness values of refined patterns. Pattern searches within selected clusters generated by PSI-Blast were performed with PRATT using default parameters. Signal peptide sequences were scanned with Signal P [24].

### Quantitative RT-PCR transcript verification

Total RNA was isolated using TRIzol® reagent (Invitrogen) or using the GE Healthcare kit (Buckinghamshire, UK) (in the case of regenerating lens tissue) according to the manufacturers' instructions. Total RNA (1 µg) was used for reverse transcription using SuperscriptII® (Invitrogen) following standard procedures. Real-time PCR was performed using the iCycler (Bio-Rad, Munich, Bayern, Germany) and ABsolute™ QPCR SYBR Green Fluorescein Mix (ABgene, Epsom, UK) or the iQ™SYBR® Green Supermix (Bio-Rad; for regenerating lens tissue). Expression levels were normalized based on RP21 or RPL27 (for regenerating lens tissue) housekeeping genes. A list of primers is supplied in Additional file 13.

### Data access

Supplemental material is available for this article. The Illumina sequence data from this study have been submitted to the NCBI Sequence read archive under accession number [ERP001353]. All assembled reads are publicly available through the Newtomics database [45] and can be downloaded at [59]. Mass spectrometry raw data and 454 reads have been uploaded to the Proteome Commons Tranche repository: A1 cell tissue [60] (hash key E9gn3jtHz9/QUGK5WlBiB+M9oP6WYjljagq0cPNCdAgUvsL3s6NAQ32Kh+RkOKtbT22c0aTyEJ4rFq+pkdY977I6VdsAAAAAAAABzA==); heart tissue [61] (hash key nSFtHPGpn+qPYPRcc3/NGKbHFpPoBh5m8fLzbkRXChOdEyGUoLguR0CTQA6F7wF/CZ0Z7jdO89t2H2hDjsxz/NzINpoAAAAAAAAB1g==); tail tissue [62] (hash key Sh9IkJHrsCQLYOeNE2sf7gNiUsxpqGWRi23/WiTHFX3dXdNGJDMcqHD9LP9JYSgRc+JmHNB3lOnTXX5B1h66cfMigEkAAAAAAAAB0g==); lens cell tissue [63] (hash key JwZJpuT9w4TPmouUz06eIVvBL726Fid+RxA8FNnbSMncIKA05OwOQEIGX8a+clxmVr7sSpo29+pnGLAmFL+PjIhj3vwAAAAAAAAB0A==); other mixed tissue [64] (hash key hPSvp96O5idgvaZy9R253/JAyYP5Qu+e/w/vej0Oq79ZDdJcmsynGVXIt/50mwx3eQDAj1UF726EmbeDtPUPDuWETb8AAAAAAAAB0A==); 454 sequencing reads [65] (hash key kdQqcmlwPViqk6Ep200pzjJnt6C73EkE5HpvvfSoAeVoK7t5iRfsC9jND+0jTUBOo6SnYZELlsh9PwMDd9RD94UYgA8AAAAAAAAB4g==).

## Abbreviations

bp: base pair; EST: expressed sequence tag; GO: Gene Ontology; LC-MS/MS: liquid chromatography coupled to a tandem mass spectrometry; NCBI: National Center for Biotechnology Information; ORF: open reading frame; SILAC: stable isotope labeling by amino acids in cell culture; UTR: untranslated region.

## Authors' contributions

ML, PAT, TBo and TB co-wrote the paper. ML, JP and MB performed the *de novo *assembly and bioinformatical analysis. KS contributed to the illumina sequencing, ML, TBo and RR contributed to the 454 and Sanger sequencing. CSM, EL and KS performed tissue preparation and RT-PCR experiments. ML, SH and MK contributed to the masspec analysis and peptide identification. MK, PAT, TBo and TB gave technical advice and contributed to the study design. All authors read and approved the final manuscript.

## Supplementary Material

Additional file 1 **Performance of different assembly strategies**. Performance comparison of four different assembly strategies comparing total number of transcripts, N50, transcripts >500 bp and transcripts >1,000 bp. Click here for file

Additional file 2 **Overall distribution of transcript annotation rate as a function of sequence length**. Transcript length (x-axis, log scale) is plotted against the percentage of overall annotation (y-axis). E-value cut-offs from e-10 to e-200 are marked in different colors. The dashed line demarks the sequence length above which transcripts were chosen for further analysis. Click here for file

Additional file 3 **Coverage of *de novo *assembled newt transcript with high quality annotations on human signaling pathways**. Fifty-eight members of the human p53 signaling pathway are matched by 47 proteins present in the assembled newt transcriptome. The use of high quality threshold criteria might have prevented detection of all family members. Click here for file

Additional file 4 **Coverage of *de novo *assembled newt transcript with high quality annotations on human signaling pathways**. The transforming growth factor beta signaling pathway containing 51 members is covered by 41 newt transcripts. Candidates identified by gene symbols are marked in pink, candidates that were not identified are marked in purple. Pathway nodes including multiple candidates that are only partially represented in the newt transcriptome are marked in dark red. Click here for file

Additional file 5 **All transcripts assigned to orthologues and corresponding length distribution with respect to the subject sequences**. Click here for file

Additional file 6 **All potential protein-coding transcripts that were validated by corresponding peptides**. The number of identified frameshifts and the total number of identified peptides is listed. The second sheet gives the example of a single candidate where peptides and alignments identified a frameshift in the nucleotide sequence. Click here for file

Additional file 7 **All identified Pfam domains in transcripts that lacked any sequence similarity to higher organisms**. The second sheet includes the domains found in candidates presented in the manuscript. Click here for file

Additional file 8 **Comparative hierarchical clustering of heart and lens expression values**. Hierarchical clustering of expressions levels in regenerating hearts (columns 1 to 9), and lenses (dorsal iris, columns 10 to 12; ventral iris, columns 13 to 15) during regeneration. Only transcripts with valid array expressions for at least 13 columns are represented. The blue cluster represents a subset of transcripts that are down-regulated at at least two stages of heart and lens regeneration. The yellow cluster marks a set of transcripts that are up-regulated during late stages of heart regeneration but lacks an obvious pattern in the regenerating lens. The red cluster represents a set of transcripts that are inversely regulated at late stages of lens regeneration but lacks an obvious pattern in the regenerating heart with the exception of a smaller subfraction that was strongly up-regulated during early heart regeneration (6 hours after heart injury). The green cluster marks a set of transcripts that are uniformly up-regulated during late stages of heart and lens regeneration. The purple cluster highlights a set of transcripts that are strongly up-regulated in regenerating lens and in the regenerating heart between 1 to 4 days after damage. All heatmap members with cluster affiliation and expression values are provided in Additional file 9. Click here for file

Additional file 9 **Expression data of all microarray spots presented in the heatmap **(Additional file 8). Click here for file

Additional file 10 **Microarray expression data of regenerating heart and lens tissues**. All candidates described in the study and represented on microarrays are shown. Click here for file

Additional file 11 **Expression of newly identified genes in regenerating newt tissues**. Real time RT-PCR analysis (n ≥ 3) of selected candidates in regenerating adult newt hearts, lenses and limbs. Values were normalized to the 0 time point and to tissues with the highest expression levels. Click here for file

Additional file 12 **Nucleotide and translated amino acid sequences of all candidate molecules described in the manuscript**. Click here for file

Additional file 13 **List of all primers used**. Click here for file
