# Supplementary figures and images for: A de novo assembly of the newt transcriptome combined with proteomic validation identifies new protein families expressed during tissue regeneration
Source: Genome Biol. 2013 Feb 20;14(2):R16. doi: 10.1186/gb-2013-14-2-r16 (PMC4054090; doi:10.1186/gb-2013-14-2-r16)

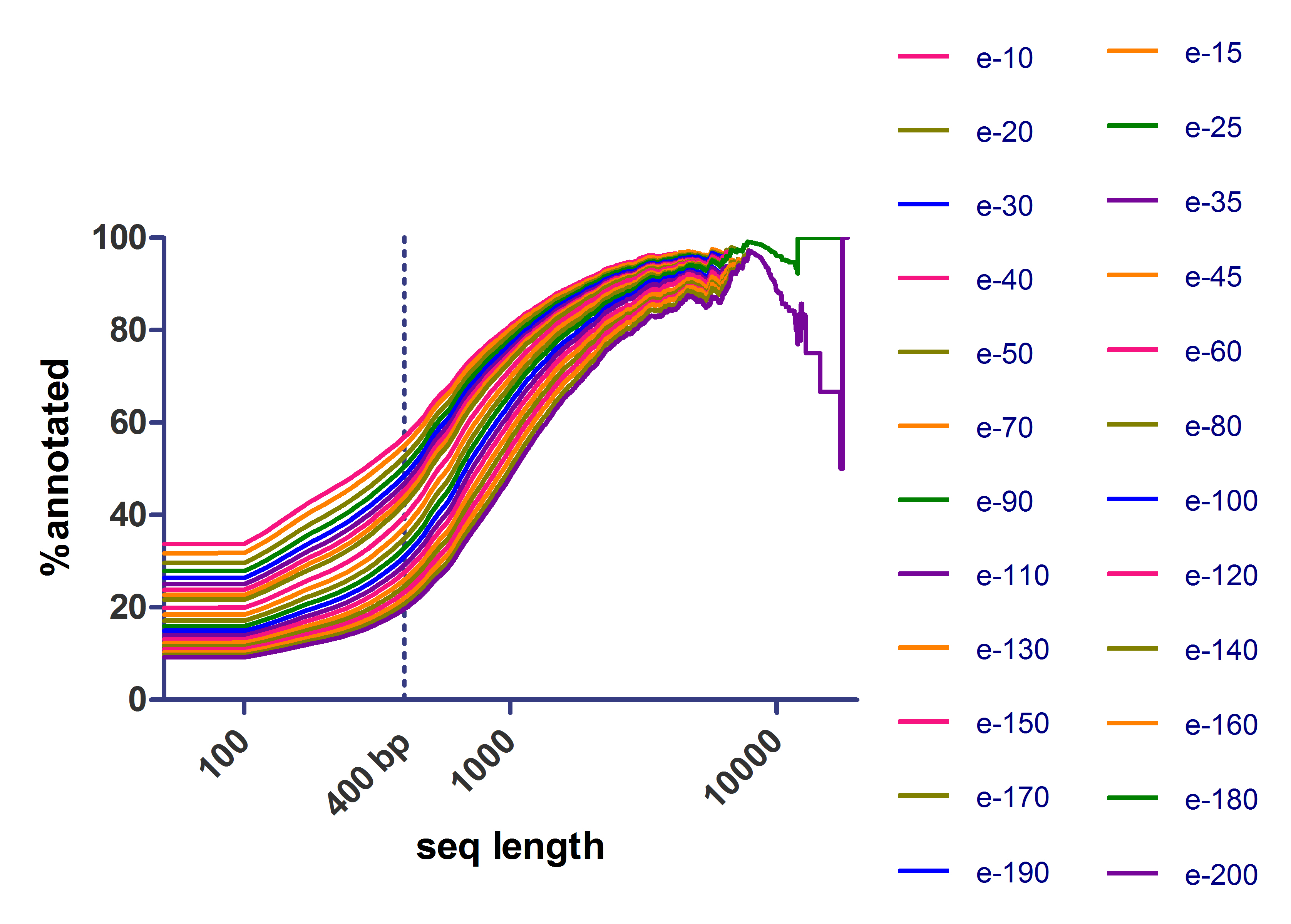

Supplement: Additional file 2 — Overall distribution of transcript annotation rate as a function of sequence length. Transcript length (x-axis, log scale) is plotted against the percentage of overall annotation (y-axis). E-value cut-offs from e-10 to e-200 are marked in different colors. The dashed line demarks the sequence length above which transcripts were chosen for further analysis. [file gb-2013-14-2-r16-S2.JPEG]

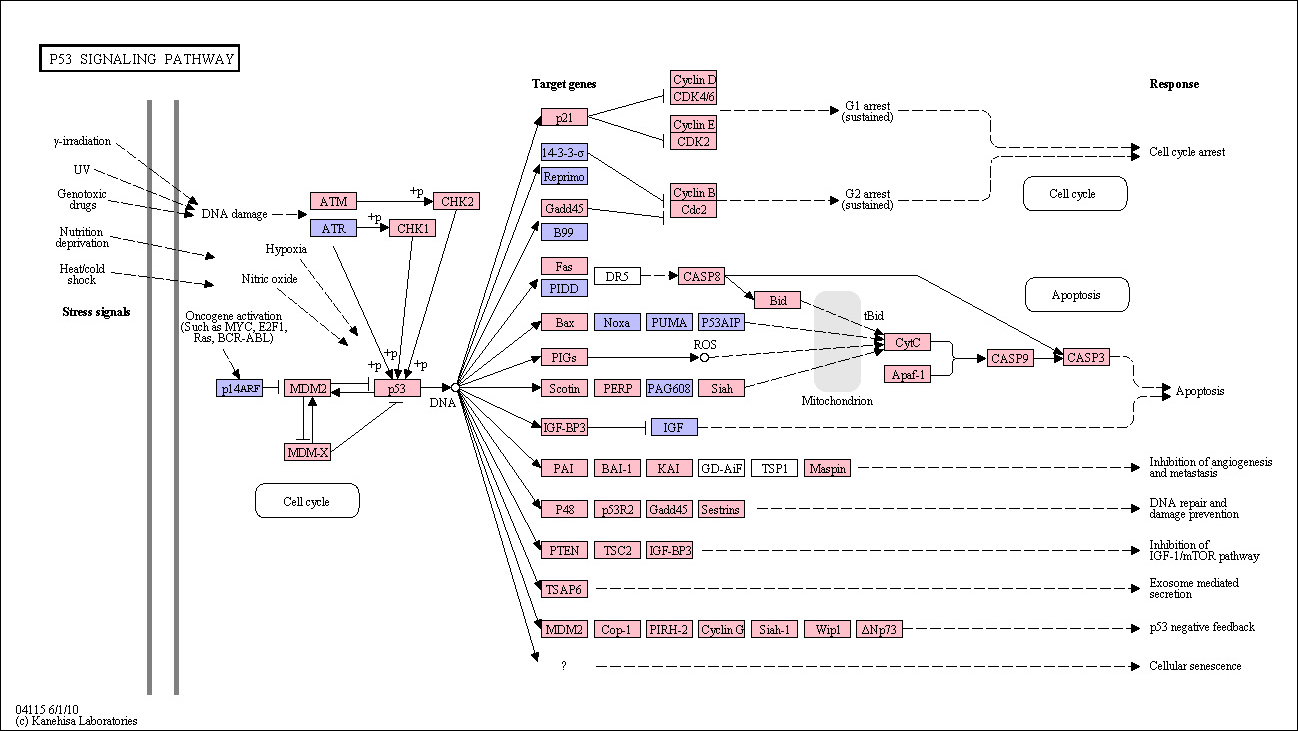

Supplement: Additional file 3 — Coverage of de novo assembled newt transcript with high quality annotations on human signaling pathways. Fifty-eight members of the human p53 signaling pathway are matched by 47 proteins present in the assembled newt transcriptome. The use of high quality threshold criteria might have prevented detection of all family members. [file gb-2013-14-2-r16-S3.JPEG]

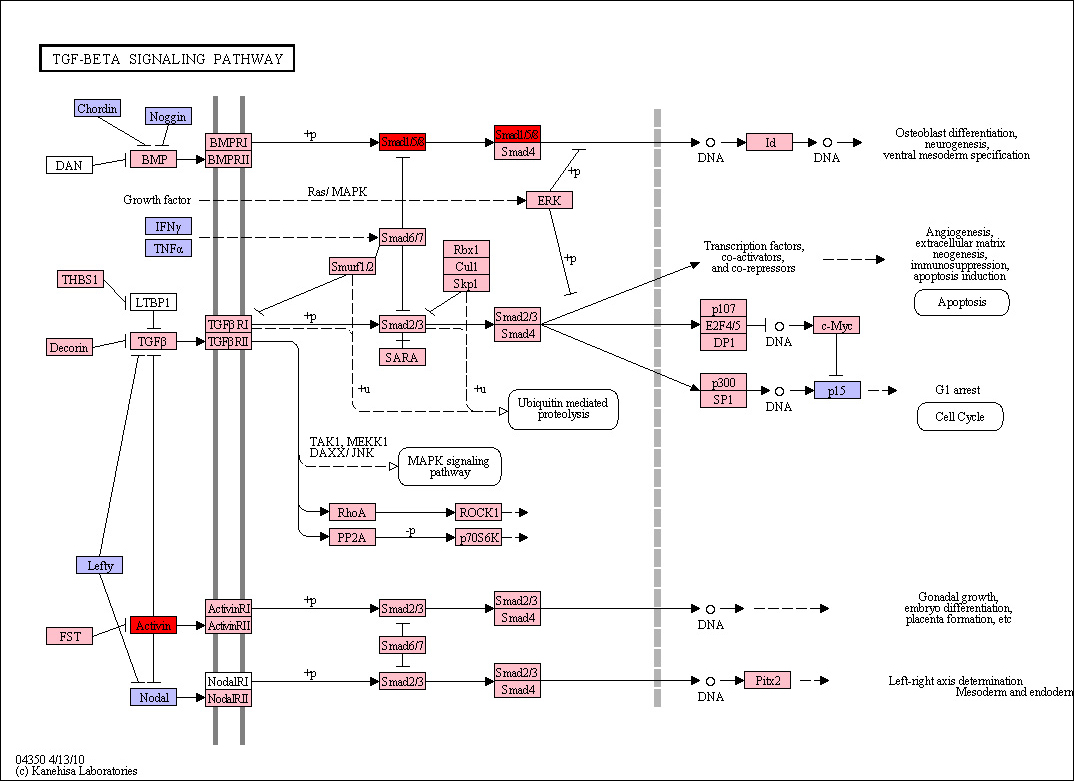

Supplement: Additional file 4 — Coverage of de novo assembled newt transcript with high quality annotations on human signaling pathways. The transforming growth factor beta signaling pathway containing 51 members is covered by 41 newt transcripts. Candidates identified by gene symbols are marked in pink, candidates that were not identified are marked in purple. Pathway nodes including multiple candidates that are only partially represented in the newt transcriptome are marked in dark red. [file gb-2013-14-2-r16-S4.JPEG]

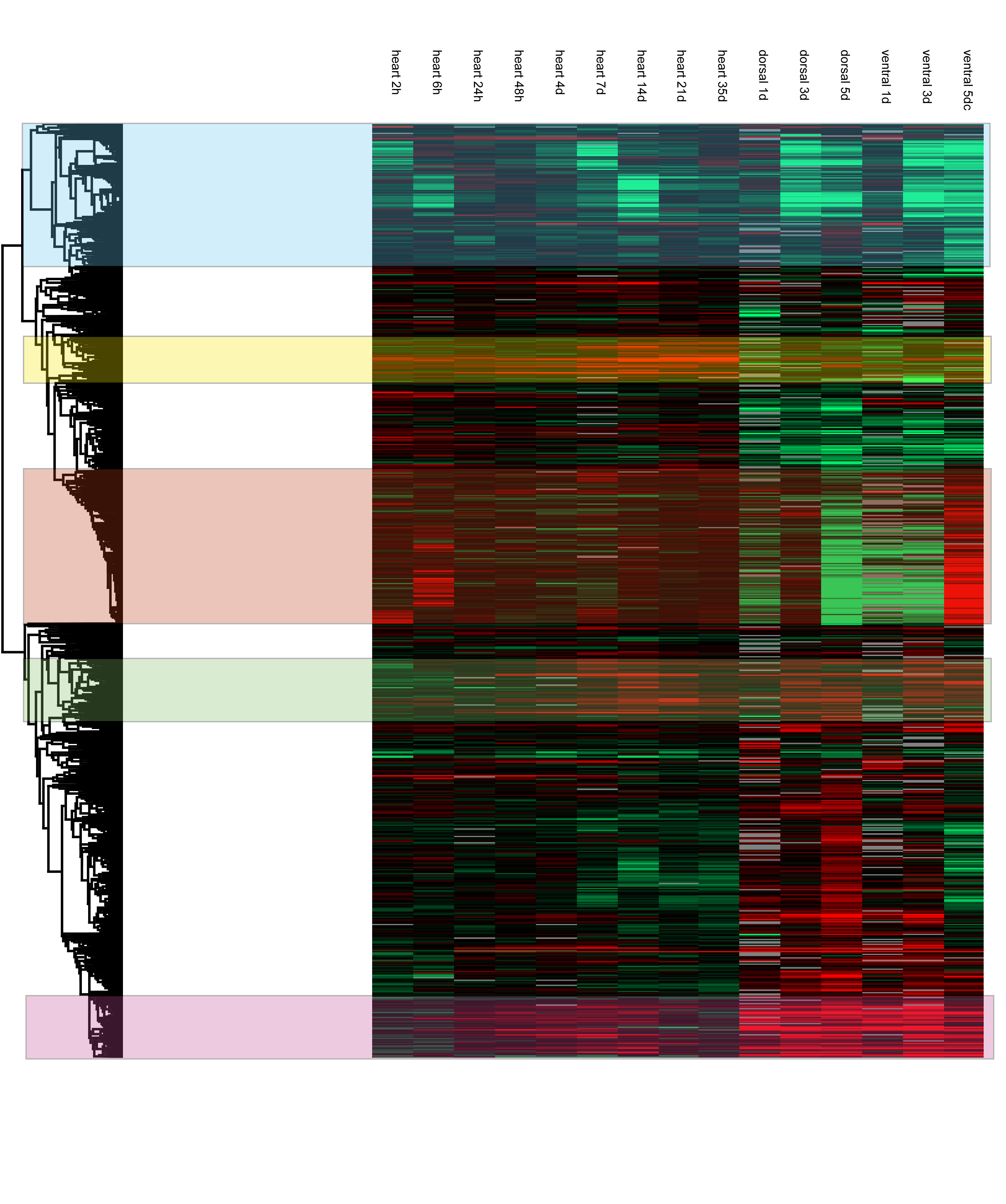

Supplement: Additional file 8 — Comparative hierarchical clustering of heart and lens expression values. Hierarchical clustering of expressions levels in regenerating hearts (columns 1 to 9), and lenses (dorsal iris, columns 10 to 12; ventral iris, columns 13 to 15) during regeneration. Only transcripts with valid array expressions for at least 13 columns are represented. The blue cluster represents a subset of transcripts that are down-regulated at at least two stages of heart and lens regeneration. The yellow cluster marks a set of transcripts that are up-regulated during late stages of heart regeneration but lacks an obvious pattern in the regenerating lens. The red cluster represents a set of transcripts that are inversely regulated at late stages of lens regeneration but lacks an obvious pattern in the regenerating heart with the exception of a smaller subfraction that was strongly up-regulated during early heart regeneration (6 hours after heart injury). The green cluster marks a set of transcripts that are uniformly up-regulated during late stages of heart and lens regeneration. The purple cluster highlights a set of transcripts that are strongly up-regulated in regenerating lens and in the regenerating heart between 1 to 4 days after damage. All heatmap members with cluster affiliation and expression values are provided in Additional file 9. [file gb-2013-14-2-r16-S8.JPEG]

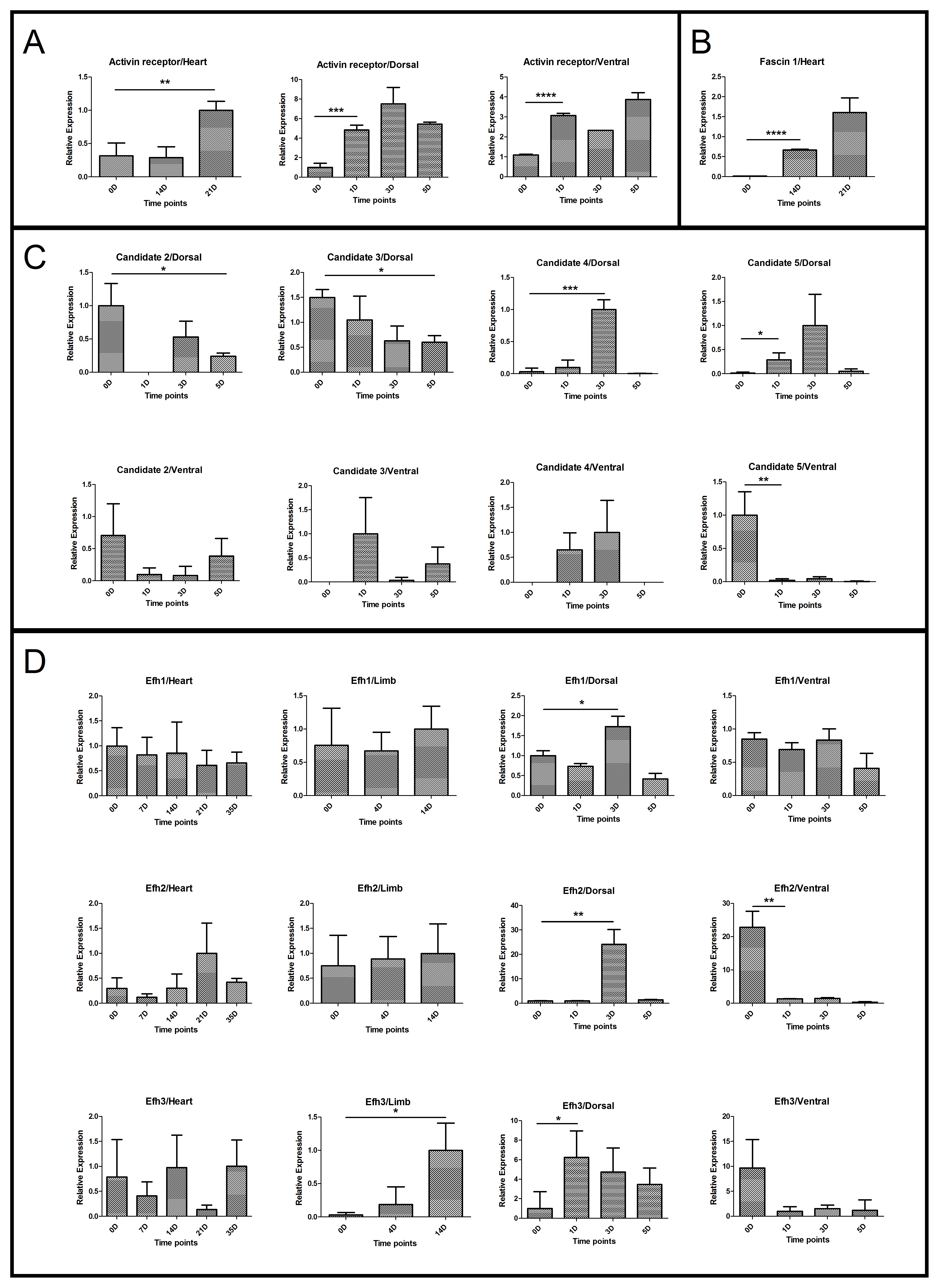

Supplement: Additional file 11 — Expression of newly identified genes in regenerating newt tissues. Real time RT-PCR analysis (n ≥ 3) of selected candidates in regenerating adult newt hearts, lenses and limbs. Values were normalized to the 0 time point and to tissues with the highest expression levels. [file gb-2013-14-2-r16-S11.JPEG]
